# Prognostic significance of TRIM28 expression in patients with breast carcinoma

**DOI:** 10.1515/med-2021-0263

**Published:** 2021-03-26

**Authors:** Wen Zhang, Zhengquan Cai, Mingzhu Kong, Anqi Wu, Zeyang Hu, Feng Wang, Hua Wang

**Affiliations:** Clinical Medicine, Medical College, Nantong University, Jiangsu, 226001, China; Department of Breast and Thyroid Surgery, Affiliated Hospital of Nantong University, Jiangsu, 226001, China; Department of Medical Imaging, Affiliated Hospital of Nantong University, Jiangsu, 226001, China; Department of Laboratory Medicine, School of Public Health, Nantong University, Jiangsu, 226019, China; Department of Laboratory Medicine, Affiliated Hospital of Nantong University, Jiangsu, 226001, China

**Keywords:** breast carcinoma, tripartite motif 28, biomarker, tumor features, prognosis

## Abstract

**Background:**

Tripartite motif 28 (TRIM28) plays a role in multiple biological functions. The expression and function of TRIM28 in breast carcinoma (BC) remain unclear. The aim of this study was to explore potential association of TRIM28 with tumor features and survival.

**Materials and methods:**

Specimens were collected from BC and adjacent normal tissues. Quantitative reverse transcription PCR (RT-qPCR) and immunohistochemistry (IHC) were performed to detect TRIM28 expression. The correlation of TRIM28 with clinicopathological features was evaluated by Chi-square test. The relationship between TRIM28 expression and survival was further analyzed by the Kaplan-Meier and Cox regression method. A receiver operating characteristic (ROC) curve was used to assess the value of TRIM28 in predicting BC.

**Results:**

In this retrospective research, it was demonstrated that TRIM28 was overexpressed in BC tissues. TRIM28 overexpression was correlated with lymph node metastasis, advanced TNM stage, and poor molecular subtype. The survival analysis showed that overall survival (OS) and progression-free survival (PFS) were significantly shorter in TRIM28-positive group. Moreover, TRIM28 was an independent prognostic factor for BC. And ROC analysis verified the diagnostic role of TRIM28 in BC.

**Conclusions:**

TRIM28 is overexpressed in BC and might be a promising prognostic and diagnostic biomarker of BC.

## 1 Introduction

Breast carcinoma (BC) is currently the most common malignancy in women worldwide. With the increasing morbidity and mortality, it has become a major public health problem [1]. At present, the mainstays of treatment are surgery, chemotherapy, endocrine therapy, radiotherapy, and targeted therapy, which to a certain extent slow the progression of BC. However, because of high heterogeneity in pathological characteristics, specific treatments are not available for some subtypes of BC. And unavoidable side effects and high cost also make the treatment quite challenging [2,3]. Additionally, various problems remain to be resolved regarding the molecular biological mechanism and treatment of BC [1]. Therefore, new biomarkers and therapeutic targets for BC are required, which would be helpful in the diagnosis and treatment of BC.

Some previous studies have found that, as a member of the family of tripartite motif (TRIM), tripartite motif 28 (TRIM28), also known as transcriptional intermediary factor 1β (TIF1β), has a role in regulating target gene transcription and DNA damage response, stimulating epithelial-mesenchymal transformation (EMT), and inducing autophagy, by binding itself to KRAB-containing zinc finger protein (KRAB-ZFP) [4,5]. Also, other researches have revealed that TRIM28 is highly expressed in various malignant tumors, such as glioma, lung cancer, and cervical cancer. Furthermore, an increasing number of researches have investigated the role of TRIM28 in BC. A recent study has confirmed that TRIM28 is a positive regulator of cell proliferation and tumor growth [6,7,8,9,10,11]. In addition, TRIM28 acts directly with TWIST1 to stabilize it, and then enhances EMT to promote breast cancer metastasis [12]. Another report verified that TRIM28 is involved in the stemness, chemotherapy resistance, and tumorigenesis in BC [13]. However, in the clinical context, the correlation between TRIM28 expression and prognosis of BC remains obscure. Hence, our purpose with this paper was to continue to explore the expression of TRIM28 in BC tissues and to analyze its association with clinicopathological parameters and prognosis of patients, in order to find out whether it is a useful target for clinical diagnosis and treatment of BC.

## 2 Materials and methods

### 2.1 Patients and tissue samples

One hundred and fourteen tissue specimens were randomly collected from the modified radical mastectomy for BC in the Affiliated Hospital of Nantong University from January 2013 to December 2014. And the corresponding specimens of adjacent tissues about 5 cm from the cancer tissue border were obtained as controls. One hundred and fourteen pairs of tissues were immediately cryopreserved in liquid nitrogen at −80°C after operation. Histopathologic specimens were all confirmed as a single primary BC by pathologists. No antitumor treatment such as surgery, chemotherapy, or radiotherapy was applied before surgery. Patients involved were all female without serious basic diseases before surgery, aged between 35 and 81 years (the median age was 52 years). Patients were followed up in our outpatient clinic or by telephone surveys, and the follow-up time was from the date of the operation to the date 5 years later or when the patient died. The last follow-up time was December 2019. The study was approved by the Ethics Committee of the Affiliated Hospital of Nantong University, and all selected patients signed an informed consent.

### 2.2 Pathological parameters of patients

The pathological parameters of patients, such as tumor size, vascular or nerve invasion condition, lymph node condition, histologic grade, TNM stage, estrogen receptor (ER), progesterone receptor (PR), Ki67, human epidermal growth factor receptor 2 (HER-2) expression levels, and molecular subtype, were recorded. The histologic grade of BC was divided into I, II, and III by the Pathology department according to the degree of differentiation from high to low [14]. TNM stage was based on the eighth edition of the breast cancer AJCC staging system [15]. The expression levels of relevant biomarkers, including ER, PR, HER-2, and Ki67, were all detected using IHC or in situ hybridization (ISH) by pathologists [16]. Ki67 expression greater than 20% was defined as positive [17]. According to the diagnosis and treatment guidelines of breast cancer in 2019 Chinese Society of Clinical Oncology (CSCO), breast cancer was divided into four kinds of molecular subtypes: Luminal A, Luminal B, HER-2-positive, and triple negative. ER, PR, and HER-2 expressions were all negative in triple-negative BC. Detailed pathological parameters are shown in Table 1.

**Table 1:** Correlations of TRIM28 expression in breast cancer tissues with clinicopathological parameters

Characteristics	TRIM28 expression		Total	*P*
	Negative (*n* = 49)	Positive (*n* = 65)		
**Age (years)**				
≤55	29	39	68	0.930
>55	20	26	46	
**Tumor size (cm)**				
≤2	24	31	55	0.892
>2	25	34	59	
**Vascular or nerve invasion**				
Positive	3	7	10	0.385
Negative	46	58	104	
**Lymph node metastasis**				
Positive	9	26	35	0.013
Negative	40	39	79	
**Histologic grade**				
Ⅰ + Ⅱ	40	44	84	0.094
Ⅲ	9	21	30	
**TNM stage**				
0 + Ⅰ	28	22	50	0.013
Ⅱ + Ⅲ	21	43	64	
**ER expression**				
Negative	14	30	44	0.056
Positive	35	35	70	
**PR expression**				
Negative	17	28	45	0.365
Positive	32	37	69	
**HER-2 expression**				
Negative	35	50	85	0.505
Positive	14	15	29	
**Molecular subtype**				
NTNBC	46	50	96	0.014
TNBC	3	15	18	
**Ki67 expression**				
Negative	34	35	69	
Positive	15	30	45	0.093

TNBC, triple-negative breast cancer; NTNBC, non-triple-negative breast cancer.

### 2.3 Quantitative real-time polymerase chain reaction assay

Approximately 80 mg of each tissue was taken, shredded, and placed in a homogenizer, and 1 mL of TRIzol reagent (Invitrogen, USA) was added for homogenization, followed by RNA extraction according to the manufacturer’s instructions. The extracted total RNA was used as a template and added with the RevertAid First Strand cDNA Synthesis Kit (Thermo Fisher Scientific, USA) according to the instruction. Then the reverse transcription was performed on the C1000 gene amplifier (BioRad, USA) to synthesize complementary DNA (cDNA). The synthesized cDNA was used as a template for real-time PCR (qPCR) and analyzed in a Roche Cobas 480 automatic fluorescent PCR analyzer (Roche Molecular Systems, Switzerland). 18S was used as an internal reference, and the relative expression level of TRIM28 was calculated with 2^−△△CT^ value. The sequence of primers used is as follows (Sangon Biological Engineering Company, Shanghai): the forward of TRIM28: TGTTTCCACCTGGACTGTCA, the reverse of TRIM28: CCAGCAGTACACGCTCACAT; the forward of 18S: GTAACCCGTTGAACCCCATT, the reverse of 18S: CCATCCAATCGGTAGTAGCG.

### 2.4 Immunohistochemical staining analysis

Formalin-fixed paraffin-embedded tissues were taken for serial section at 5 µm thickness by LEICA RM2035 microtome (Leica, Germany). All sections were deparaffinized in xylene and rehydrated with gradient ethanol, followed by rinsing in phosphate-buffered saline (PBS) buffer. Slides were heated in the boiled EDTA antigen retrieval buffer (PH 9.0) for 18 min and then cooled to the room temperature. A PAP Pen was used to drawn a circle at the tissue margin. And 100 µL of 3% H_2_O_2_ was added for blocking endogenous peroxidase. After incubation at room temperature for 15 min, PBS was used for rinsing slides again. The rabbit antihuman TRIM28 polyclonal antibody (1:100, Abcam, UK) was added to cover tissues, and then incubated with PBS for 16 h at 4°C. The goat anti-rabbit IgG labeled with Biotin (DAKO, Denmark) was added to tissues and incubated at room temperature for 20 min. Slides were put into DAB color developing solution (1:20) (Invitrogen, USA), and the color development degree was observed under BX51 microscope (Olympus, Japan). After that, hematoxylin counterstaining and hydrochloric acid ethanol differentiation were performed for 10 and 3 s, respectively. Having been dehydrated with gradient ethanol, slides were placed in xylene. Last, Neutral resin was used to seal tissue sections. PBS was utilized as a negative control of the primary antibody.

### 2.5 Immunohistochemical scoring

The signal of TRIM28 was mainly expressed in the nucleus. And brown-yellow particles being emerging in the nucleus, it was judged as positive. The expression level of TRIM28 was evaluated by the percentage and staining intensity of positive cells. Randomly select three high-magnification microscope fields, calculate the percentage of positive cells in each field, and then calculate the average number, which was the required percentage of positive cells. The percentage scores of positive cells were as follows:1 (0–25%), 2 (26–50%), 3 (51–75%), and 4 (76–100%). The positive cell staining intensity scores were as follows: 0 (negative), 1 (weak positive), 2 (medium positive), and 3 (strong positive). The percentage of positive cells multiplied by the staining intensity score was the total immunohistochemical score. The mean value (5.66) of immunohistochemical score was used as cutoff. Those with a score ≥6 were defined as positive.

### 2.6 Statistical analysis

SPSS 25.0 and GraphPad Prism 8.0 software were used for statistical analysis. All quantitative data were expressed by \bar{\chi }\pm s], and analysis of variance and *t* test were used for comparison between groups. All categorical data were expressed by frequencies and rates; *χ* ^*2*^ test was used for comparison between groups. The prognosis of BC patients was evaluated by univariate and multivariate Cox proportional hazards regression models. Kaplan-Meier method was used for survival curve analysis, and differences were compared by Log-Rank test. By using the ROC curve analysis, the area under the curve (AUC) was estimated to assess the diagnostic role of TRIM28 in BC cases. The Optimal Cutoff Value, sensitivity, and specificity were obtained by the calculation of the highest Youden index (the sum of sensitivity and specificity minus 1). The corresponding figure of the ROC curve was plotted by the software of SPSS 25.0. *P* value less than 0.05 was defined as statistically significant.

## 3 Results

### 3.1 Correlation between TRIM28 expression and clinical features of BC

A total of 114 breast cancer patients were enrolled in this study, in order to analyze the relationship between TRIM28 expression level and clinicopathological parameters, including age, tumor size, vascular or nerve invasion condition, lymph node condition, histologic grade, TNM stage, ER, PR, Ki67, HER-2 expressions, and molecular subtype. According to the result of IHC, patients were divided into TRIM28-positive group and TRIM28-negative group. Highly expressed TRIM28 in BC was associated with lymph node metastasis (*P* = 0.013), higher TNM stage (*P* = 0.013), and worse molecular subtype (*P* = 0.014). Meanwhile, TRIM28 expression was not significantly related to age, tumor size, vascular or nerve invasion condition, histologic grade, ER, PR, HER-2, and Ki67 expressions (Table 1).

### 3.2 TRIM28 overexpression in BC

**Figure 1:**
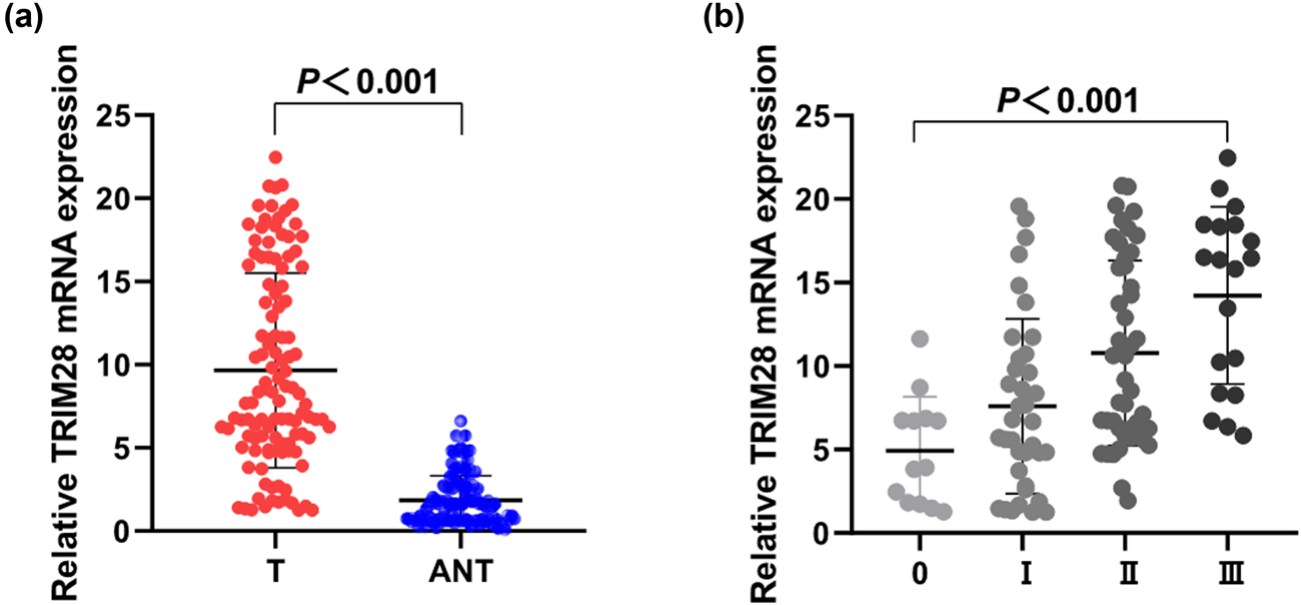
The analysis of TRIM28 expression by PCR. (a) TRIM28 expression in BC tissues was significantly higher than that in adjacent normal tissues (*P* < 0.001). (b) TRIM28 expression in different TNM stages (*P* < 0.001). ANT, adjacent normal tissue; T, tumor.

In RT-qPCR, the expression of TRIM28 mRNA was detected in 114 BC and paired adjacent tissues. The results demonstrated that TRIM28 mRNA was significantly increased in BC tissues than adjacent tissues (*P* < 0.001, Figure 1a). And the overexpression of TRIM28 in cancer tissues was significantly correlated with advanced TNM stages (*P* < 0.001, Figure 1b). However, there was no significant difference in TRIM28 expression between different molecular subtypes (*P* = 0.105).

**Figure 2:**
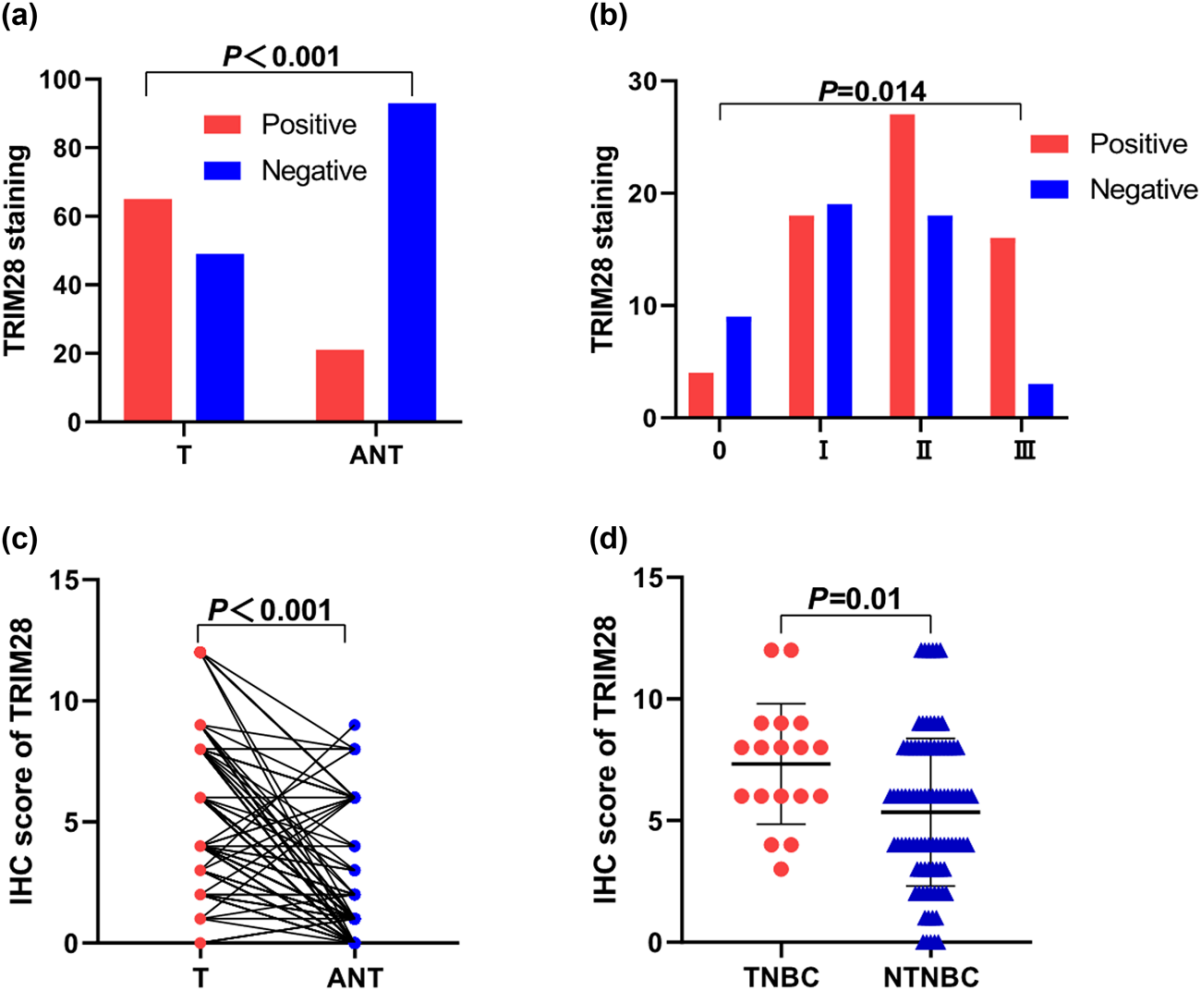
The IHC staining of TRIM28 expression in tissues. (a) The comparison of the percentage of positive TRIM28 staining in tissues between tumors and ANTs (*P* < 0.001). (b) The distribution of positive TRIM28 staining at different TNM stages (*P* = 0.014). (c) The IHC score of TRIM28 in cancer and adjacent tissues (*P* < 0.001). (d) The IHC score of TRIM28 in triple-negative breast cancer and non-triple-negative breast cancer (*P* = 0.01). ANT: adjacent normal tissue; T: tumor; TNBC: triple-negative breast cancer; NTNBC: non-triple-negative breast cancer.

**Figure 3:**
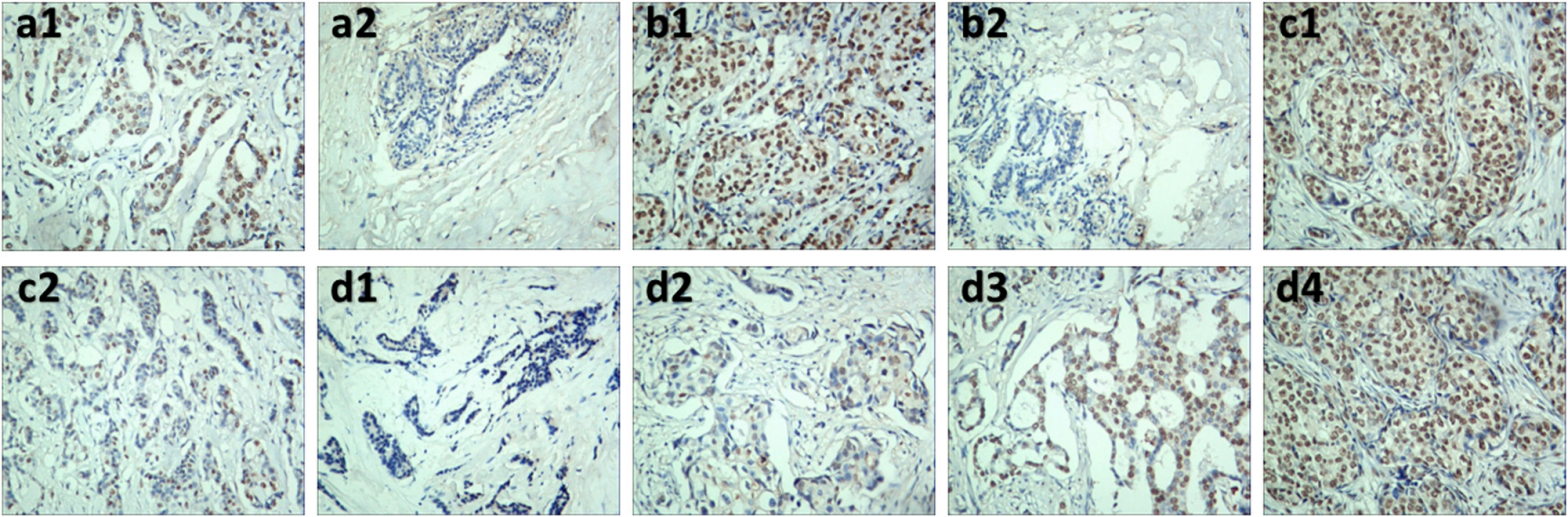
Representative IHC staining of TRIM28 in BC tissues and corresponding tissues adjacent to cancer (IHC ×200). a1–b2: TRIM28 expression in two groups of BC and adjacent tissues, a1 and a2 were in the first group, b1 and b2 were in the second group, a1 and b1 were cases of breast cancer tissues, a2 and b2 were cases of adjacent tissues. c1 and c2: c1 was a case of TRIM28 expression in triple-negative breast cancer, and c2 was a case of TRIM28 expression in non-triple-negative breast cancer. d1–d4: TRIM28 expression in different TNM stages, d1 was at stage 0, d2 was at stage I, d3 was at stage II, d4 was at stage III.

To explore the expression of TRIM28 in different TNM stages, IHC was carried out for further analysis. The results showed that TRIM28 was mainly located in the nucleus. There were 65 cases (57.0%) with positive expression of TRIM28 in BC tissues, and 21 cases (18.4%) with positive expression of TRIM28 in adjacent tissues. The difference was statistically significant (*P* < 0.001, Figure 2a). The immunohistochemical score of BC tissues was significantly higher than that of adjacent cancer tissues (*P* < 0.001, Figure 2c). Of all 114 samples, 18 (15.8%) were triple-negative breast cancer, whose immunohistochemical score was significantly higher than that of non-triple-negative breast cancer (*P* = 0.01, Figure 2d), suggesting that the expression level of TRIM28 was associated with molecular subtypes in BC. Additionally, among 114 BC tissues, there were 13 cases at stage 0 (11.4%), 37 cases at I (32.5%), 45 cases at II (39.5%), and 19 cases at III (16.7%). The number of TRIM28 overexpression in all cases were 4 (30.8%) at stage 0, 18 (48.6%) at I, 27 (60.0%) at II, and 16 (84.2%) at III. TRIM28 expression gradually increased with TNM stages. Most tissues at advanced stages had stronger staining (Figure 2b). Some representative immunohistochemical images were shown below (Figure 3a–d).

### 3.3 Prognostic significance of TRIM28 in BC patients

**Figure 4:**
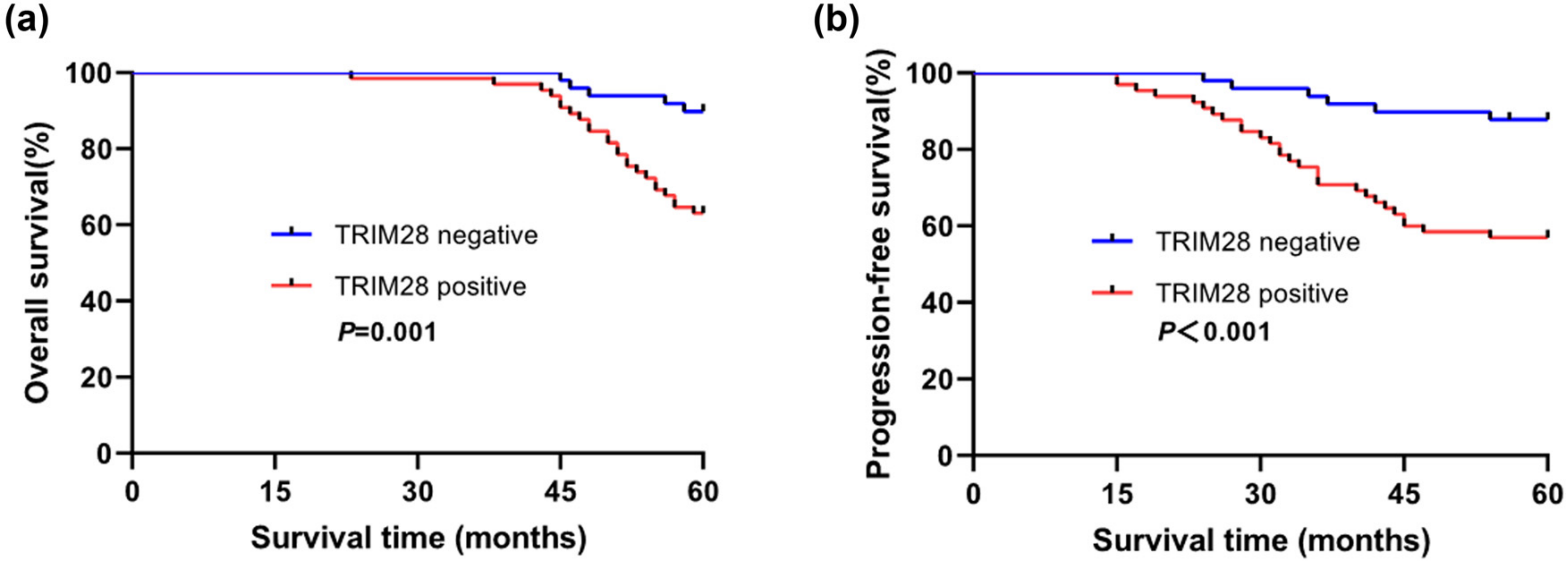
Kaplan-Meier survival analysis of high TRIM28 expression (*n* = 49) and low TRIM28 expression (*n* = 65) in BC patients. (a) The relationship between TRIM28 expression and overall survival rate (*P* = 0.001); (b) The relationship between TRIM28 expression and progression-free survival rate (*P* < 0.001).

In this study, the relationship between TRIM28 expression and BC prognosis was also evaluated. Kaplan-Meier method was performed to analyze the association between overall survival (OS), progression-free survival (PFS), and TRIM28 expression in BC patients. As shown in Figure 4, TRIM28-positive group (*n* = 65) showed shorter OS time (Figure 4a, *P* = 0.001) and PFS time (Figure 4b, *P* < 0.001) than those of the negative group (*n* = 49).

Furthermore, Cox proportional hazards regression model was used to assess the prognostic value of TRIM28 for BC. Because of their role as potential risk factors for poor prognosis, TRIM28 expression, age, tumor size, vascular or nerve invasion condition, lymph node condition, histologic grade, TNM stage, ER, PR, HER-2, Ki67 expression, and molecular subtype were selected as risk variables. In univariate and multivariate analysis, molecular subtype and TRIM28 expression were independent risk factors regarding OS (Table 2, HR = 0.105, 95% CI = 0.017–0.665, *P* = 0.017; HR = 3.061, 95% CI = 1.008–9.297, *P* = 0.048). TRIM28 and HER-2 expression, as well as molecular subtype, were independent risk factors for PFS (Table 3, HR = 3.719, 95% CI = 1.406–9.838, *P* = 0.008; HR = 3.136, 95% CI = 1.039–9.466, *P* = 0.043; HR = 0.179, 95% CI = 0.037–0.879, *P* = 0.034).

**Table 2:** Univariate and multivariate Cox regression analysis of overall survival in 114 breast cancer patients

Variables	OS			
	Univariate *P*	Multivariate		
		Hazard ratio	95% confidence interval	*P*
Age (years) (≤55 vs >55)	0.722	0.863	0.374–1.995	0.731
Tumor size (cm) (≤2 vs >2)	0.071	1.787	0.605–5.279	0.294
Vascular or nerve invasion (positive vs negative)	0.063	0.929	0.310–2.781	0.895
Lymph node metastasis (positive vs negative)	<0.001	2.714	0.944–7.801	0.064
Histologic grade (Ⅰ + Ⅱ vs Ⅲ)	0.272	1.534	0.648–3.630	0.330
TNM stage (0 + I vs II + III)	0.001	2.134	0.418–10.879	0.362
ER (negative vs positive)	0.007	3.001	0.510–17.665	0.224
PR (negative vs positive)	0.004	0.519	0.103–2.606	0.426
HER-2 (negative vs positive)	0.579	2.104	0.598–7.403	0.247
Molecular subtype (TNBC vs NTNBC)	<0.001	0.105	0.017–0.665	0.017
Ki67 (negative vs positive)	<0.001	2.364	0.952–5.868	0.064
TRIM28 (negative vs positive)	0.003	3.061	1.008–9.297	0.048

TNBC, triple-negative breast cancer; NTNBC, non-triple-negative breast cancer.

**Table 3:** Univariate and multivariate Cox regression analysis of progression-free survival in 114 breast cancer patients

Variables	PFS			
	Univariate *P*	Multivariate		
		Hazard ratio	95% confidence interval	*P*
Age (years) (≤55 vs >55)	0.394	0.615	0.278–1.362	0.231
Tumor size (cm) (≤2 vs >2)	0.059	1.769	0.631–4.956	0.278
Vascular or nerve invasion (positive vs negative)	0.019	1.184	0.408–3.432	0.756
Lymph node metastasis (positive vs negative)	0.001	2.074	0.727–5.917	0.173
Histologic grade (Ⅰ + Ⅱ vs Ⅲ)	0.295	1.564	0.704–3.473	0.272
TNM stage (0 + I vs II + III)	0.001	2.004	0.499–8.044	0.327
ER (negative vs positive)	0.015	3.111	0.689–14.039	0.140
PR (negative vs positive)	0.011	0.488	0.118–2.023	0.323
HER-2 (negative vs positive)	0.418	3.136	1.039–9.466	0.043
Molecular subtype (TNBC vs NTNBC)	<0.001	0.179	0.037–0.879	0.034
Ki67 (negative vs positive)	0.001	1.759	0.798–3.878	0.162
TRIM28 (negative vs positive)	0.001	3.719	1.406–9.838	0.008

TNBC, triple-negative breast cancer; NTNBC, non-triple-negative breast cancer.

**Figure 5:**
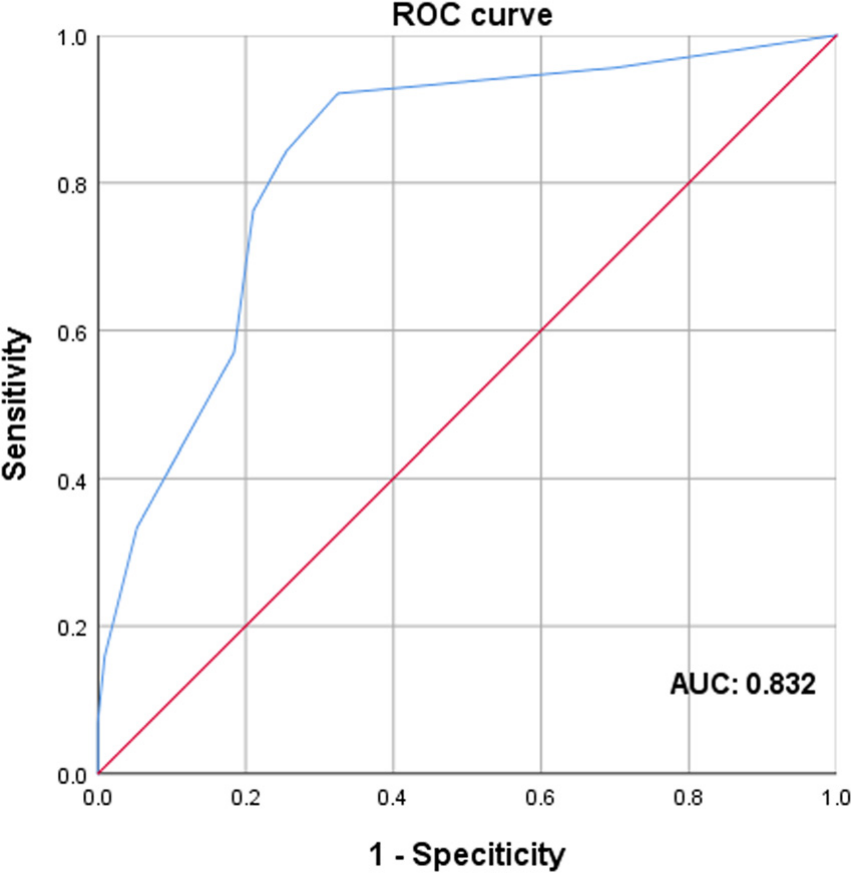
ROC curve analysis to detect the predictive value of TRIM28 in BC patients.

Finally, in order to explore the diagnostic usefulness of TRIM28 in BC, IHC scores in 114 cases of breast cancer tissues and the corresponding adjacent normal tissues were included in the study. From the ROC curve analysis, it can be proved that the expression level of TRIM28 can distinguish breast cancer tissues from normal ones. The AUC was 0.832, with the sensitivity of 0.921, the specificity of 0.675, and the 95% confidence interval of 0.777–0.886 (Figure 5).

## 4 Discussion

Due to the high heterogeneity of BC, personalized medicine is continuously being explored in order to improve patients’ prognosis. The two main problems in the research of BC are the treatment of highly aggressive triple-negative BC and chemotherapy-resistant subgroups [18]. The TRIM family, consisting of a RING domain, 1 or 2 B-box domains, and a coiled-coil domain, has been found to be highly expressed in the nucleus. They are considered as transcriptional regulators that regulate chromatin formation and play a vital role in the process of intracellular signal transduction, development, apoptosis, protein quality control, innate immunity, autophagy, carcinogenesis, etc. [5]. In recent years, the biological role of TRIM28 in cancer cells has come into public attention [19]. It is highly expressed in embryonic stem cells and various tumors and is ubiquitous in tumor development [20]. In prostate cancer, as a key upstream regulator of TRIM24, TRIM28 was proved to interact with TRIM24 to prevent its ubiquitination and degradation by SPOP and also enhance the signal transduction of Androgen receptor (AR). TRIM28 promoted the proliferation of prostate cancer cells and was upregulated in aggressive prostate cancer, leading to poor clinical prognosis [21]. In a study of cervical cancer, it was demonstrated that TRIM28 promoted the growth of cervical cancer cells by activating the mTOR signaling pathway. The utilization of mTOR inhibitors could reduce TRIM28-induced cell proliferation, suggesting that it could be used as a potential therapeutic target for cervical cancer [8]. Fitzgerald S et al. [22] analyzed the TRIM28-related pathways in interstitial fibroblasts and epithelial tumor cells through comprehensive proteomics analysis of the molecular network in the tumor microenvironment, showing that TRIM28 had a predictive role in the prognosis of colorectal cancer. Also, TRIM28 has been certified to be vital for activating autophagy and promoting cell proliferation in glioma [23]. These researches above have shown that TRIM28 strongly affects the occurrence and development of multiple carcinomas. But currently, in the clinical context, TRIM28 expression in BC and its correlation with clinical parameters remain unrecognized, and the association between TRIM28 and the prognosis of BC patients has not been elucidated.

In this study, the expression level of TRIM28 in 114 samples was analyzed by RT-qPCR and IHC. The results showed that TRIM28 functioned as a cancer-promoting gene in BC. In RT-qPCR and IHC, the expression of TRIM28 in BC tissues was significantly higher than that in adjacent tissues, and with the TNM stage increasing, its expression level also increased gradually. Different from what was expected before, no significant difference was detected in the expression of TRIM28 between BC subtypes via RT-qPCR, which might result from the small sample size. In addition, by analyzing the correlation between TRIM28 expression and clinicopathological parameters of BC in IHC, it was suggested that higher TRIM28 expression level predicted worse lymph node condition, worse molecular subtype, and more advanced TNM stage. Furthermore, the data above indicated that TRIM28 was positively correlated with the malignancy of BC. Next, by Kaplan-Meier method and Log-Rank test, it was found that the high expression of TRIM28 predicted a shorter OS and PFS. Also, via Cox proportional hazards regression analysis, it was concluded that molecular subtype and TRIM28 expression were significantly related to postoperative OS, while molecular subtype, HER-2, and TRIM28 expression were associated with postoperative PFS. All of the above confirmed that TRIM28 and poor molecular subtype – triple-negative breast cancer (TNBC), were independent risk factors for poor prognosis of BC. And ROC curve analysis further demonstrated the diagnostic and predictive value of TRIM28 in BC patients. In summary, the expressions of TRIM28 mRNA and protein were substantially similar in BC. The method of integrating RT-qPCR with IHC might be clinically helpful for the examination and diagnosis of BC.

Some limitations exist in this research. It was a retrospective study with a relatively small sample size. Recruited patients who met indications for operative intervention were at relatively early stages, so no metastatic BC case was enrolled. Also, because BC patients were dominated by females, male patients were not involved. These samples were composed largely of invasive carcinomas. Consequently, no further comparison was made between different pathological types. Diverse postoperative adjuvant therapies might make a difference to prognosis, which to some extent influenced the outcome of this study. Furthermore, a series of researches on function and mechanism are required to verify the effect of TRIM28 on the migration and proliferation of BC cells and to explore the upstream and downstream molecular mechanisms of TRIM28 in promoting breast cancer progression.

In short, this was the first report to analyze the association between the expression status of TRIM28 and corresponding clinicopathological characteristics in BC, proving that TRIM28 is positively correlated with the malignancy of BC. As an independent risk factor for BC, TRIM28 may represent a predictive and prognostic biomarker and is expected to become a new target for the diagnosis and treatment of BC.
